# Supplementary material for: Engineered factor Xa variants retain procoagulant activity independent of direct factor Xa inhibitors
Source: Nat Commun. 2017 Sep 13;8:528. doi: 10.1038/s41467-017-00647-9 (PMC5597622; doi:10.1038/s41467-017-00647-9)
Supplement: Supplementary file 1 — Supplementary Information [file 41467_2017_647_MOESM1_ESM.pdf]

File Name: Supplementary Information
Description: Supplementary Figures, Supplementary Tables and Supplementary Reference

File Name: Peer Review File

Supplementary Figures and Legends

```
                                                  99         143  146          174          189 - 192  195            213 215 217 219        227 228
1  H.sapiens       < H91NRFT-------------KETYDFD> <GFGRTHEKGR> <SSSFIIT> <KQEDACQGDSGGP> <TGIVSWGEGCARKGKYGIYTKV231
2  M.musculus      < H91NKFQ-------------RDTYDYD> <GFGRTHEKGR> <STSFSIT> <KLEDACQGDSGGP> <TGIVSWGEGCARKGKYGIYTKV231
3  B.taurus        < H91SRFV-------------KETYDFD> <GFGRTHEKGR> <SSSFTIT> <QPEDACQGDSGGP> <TGIVSWGEGCARKGKFGVYTKV231
4  S.scrofa        < H91SKFV-------------RETYDFD> <GFGRTHERGR> <SSSFLIT> <QPEDACQGDSGGP> <TGIVSWGEGCARRGKYGVYTKV231
5  G.gallus        < H91SKYI-------------AETYDND> <GFGREFEAGR> <STNFAIT> <EQKDACQGDSGGP> <TGIVSWGEGCARKGKYGVYTKL231
6  M.gallopavo     < H91SKYI-------------AETYDND> <GFGREFEAGR> <STNFAIT> <EQKDACQGDSGGP> <TGIVSWGEGCARKGKYGIYTKL231
7  X.tropicalis    < H91PRFV-------------KSTYDYD> <GFGRIHERGR> <SSTFAIT> <EVKDACQGDSGGP> <TGIVSWGEGCARKGKFGVYTKV231
8  A.carolinensis  < H91QKFV-------------LATYDYD> <GFGRLHERGR> <SSNFVIT> <LAQDACQGDSGGP> <TGVISWGEGCAREDKYGIYTKV231
9  P.textilis      < H91QKFVP-------------ATYDYD> <GFGRTRERGK> <SSNFPIT> <LPQDACQGDSGGP> <TGIVSWGEGCAQTGKYGVYTKV231
10 T.carinatus     < H91QKFVP-------------STYDYD> <GFGRTRERGQ> <SSNFPIT> <LPQDACQGDSGGP> <TGIISWGEGCAQTGKYGAYTKV231
11 P.textilis  1   < H91KKFVPPQKAY----KFDLAAYDYD> <GFGRIFEKGP> <SSETPIT> <LPRDACQGDSGGP> <TGIVSSGEGCARNGKYGIYTKL231
12 P.textilis  2   < H91KKFVPPKKSQEFYEKFDLVSYDYD> <GFGGIFERGP> <SSNFPIT> <LPQDACQGDSGGP> <TGIVSWGEGCARKGRYGIYTKL231
13 T.carinatus 3   < H91TKFVPPNYYYVHQN-FDRVAYDYD> <GFGRIQFKQP> <SSDFRIT> <LPQDACQGDSGGP> <TGIISWGEGCARKGKYGVYTKV231
14 D.rerio         < H91KNYQP-------------DTYHND> <GFGRVREGGL> <SSNFKIS> <EEKDACQGDSGGP> <TGVVSWGEGCARKGKYGVYTQV231
15 T.rubripes      < H91YNYKP-------------NTYHND> <GFGRLGGNRQ> <STSLRIS> <IAKDACQGDSGGP> <TGIVSWGEGCAQKGKYGVYTQV231
```

Supplementary Fig. 1. Alignment of factor X serine protease domain sequences. Serine protease domain regions of FX were aligned to corresponding FX regions from several selected vertebrates. The His91-Val231 sequence is shown with the non-conserved 99-loop residues specific for *P. textilis* isoform FX[1], *P. textilis* venom FX[2], and *T. carinatus* venom FX[3] in bold [1]. Residues with the shortest Minimal Interatomic distances from their side chain to apixaban as observed in the MD simulation of apixaban-bound human FXa and as indicated in **Fig. 1A** are numbered and indicated in bold (Y99, R143, E146, F174, D189, A190, C191, Q192, S195, V213, W215, E217, C219, I227, Y228).

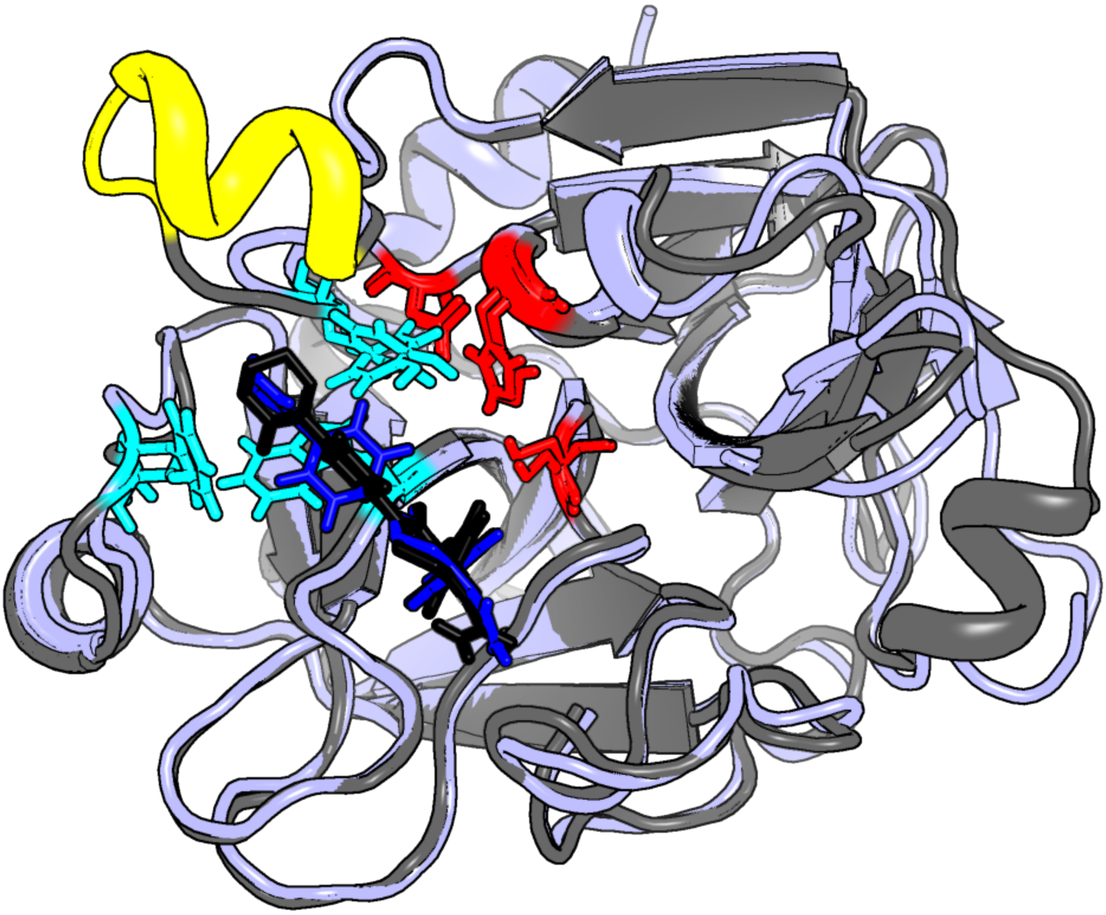

Supplementary Fig. 2. Apixaban binding orientation in human and snake factor Xa. Apixaban (blue sticks) bound to human FXa (light blue; PDB ID 2P16) was overlaid with apixaban (black sticks) docked into *P. textilis* isoform FXa (grey; PDB ID 4BXW). The isoform FXa insertion region PQKAYKFDL is highlighted in yellow, red residues indicate the position of the catalytic triad in both proteases, and cyan residues indicate the position of the S4 subsite residues Tyr99, Phe174, and Trp215.

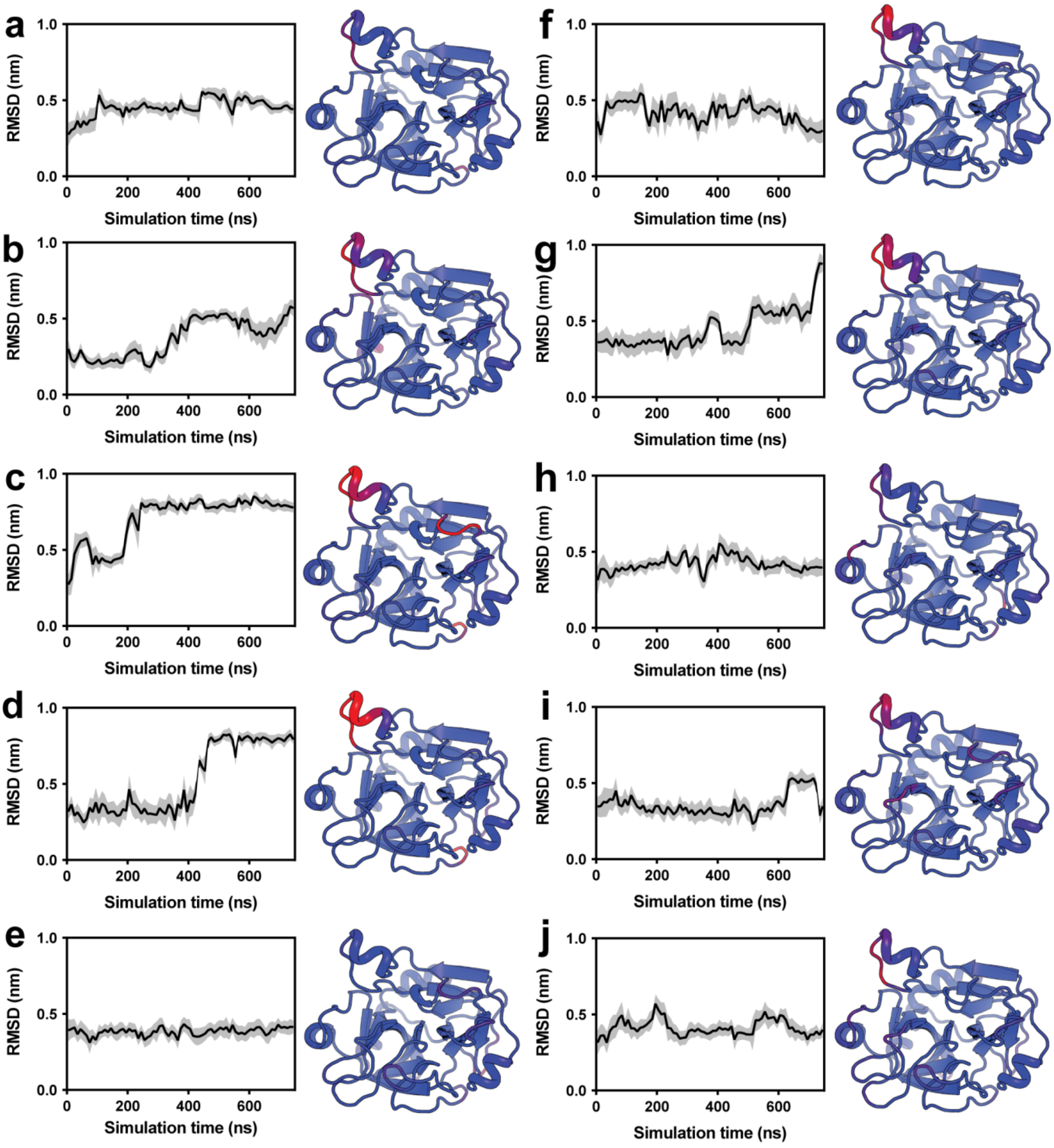

Supplementary Fig. 3. Molecular Dynamics simulations of apixaban-bound and unbound snake isoform factor Xa. Root-mean-square deviations **(**RMSD) of the backbone positions in the isoform FXa extended 99-loop region PQKAYKFDL during five independent 750 ns MD simulations of apixaban-bound (APX bound) (*a-e*) or unbound (apo) (*f-j*) isoform FXa are presented as block averages over 10 ns intervals. The corresponding single standard deviation interval is indicated (grey

density). The local flexibility of the *P. textilis* isoform FXa main-chain atoms was evaluated and calculated using GROMACS tool *g_rmsf* from atomic root-mean-square fluctuations. The resulting Debye-Waller factors (or B-factors) are projected onto the initial conformation of *P. textilis* isoform FXa (PDB ID 4BXW) as a blue-red gradient representing a ≤10 Å^2 to ≥300 Å^2 range of mobility. The B-factors are displayed for independent MD simulations starting from the same atomic coordinates but different randomly assigned velocities. The 750 ns MD simulations of apixaban-bound isoform FXa (*a-e*) correspond to simulations #1-5 in **Fig. 3a-e** and **Supplementary Fig. 4a-e**.

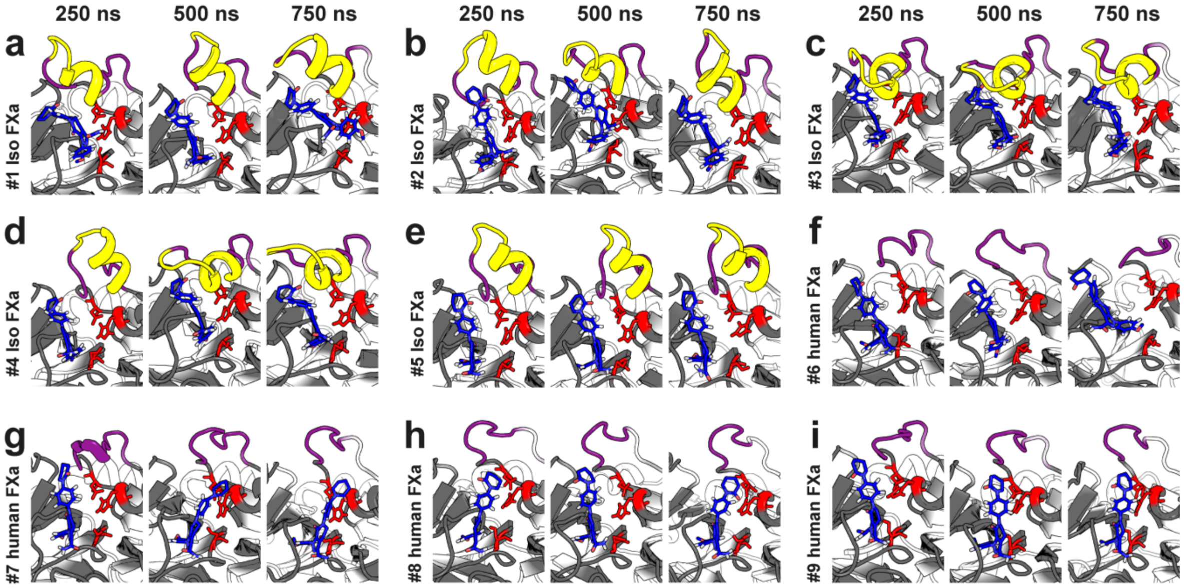

Supplementary Fig. 4. Factor Xa conformations during Molecular Dynamics simulations. The conformations of the isoform FXa-apixaban complex (simulations #1-5; *a-e*) and human FXa (simulations #6-9; *f-i*) at 250, 500, and 750 ns of MD simulations are depicted for each independent simulation. Apixaban (blue), the 99-loop (magenta), the isoform FXa extended 99-loop region PQKAYKFDL (yellow), and the catalytic triad residues (red) are highlighted. The five independent simulations of apixaban-bound isoform FXa (*a-e*) correspond to simulations #1-5 in **Fig. 3a-e** and **Supplementary Fig. 3a-e**. The four independent simulations of human FXa (*F-I*) correspond to simulations #6-9 in **Fig. 3f-i**.

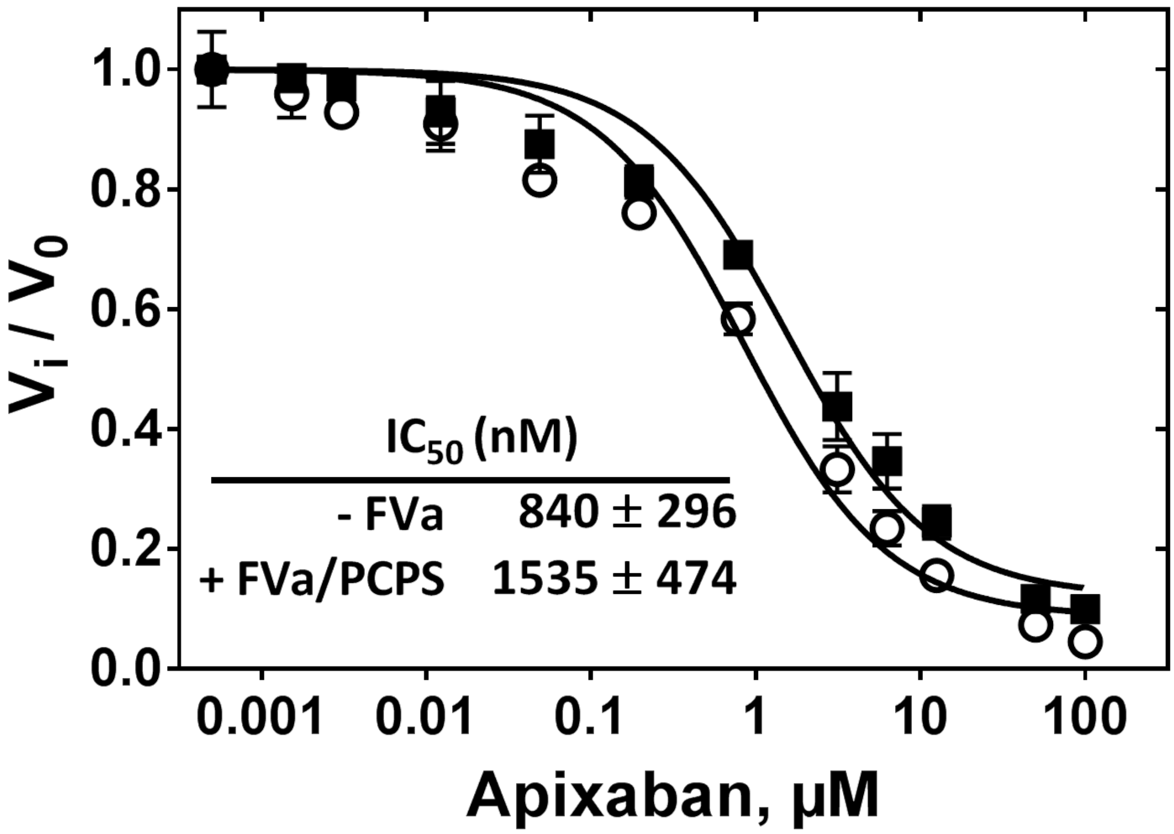

Supplementary Fig. 5. Inhibition of factor Xa-C by apixaban in the absence and presence of the cofactor Va. The rate of peptidyl substrate conversion (250 µM SpecXa) by free (open circles) or prothrombinase-assembled (PCPS, 50 µM ; FV-810 (FVa, 30 nM; closed squares) purified recombinant human FXa-C (5 nM) was determined in the absence (V_0) or presence (V_i) of increasing concentrations (0.002 – 100 µM) of the inhibitor apixaban. The lines were drawn following nonlinear regression analysis of the data sets, and the fitted parameters for IC_{50} ± 1 standard deviation of the induced fit are shown in the inset. The data are the means of three independent experiments, and are normalized for activity in the absence of inhibitor (V_0).

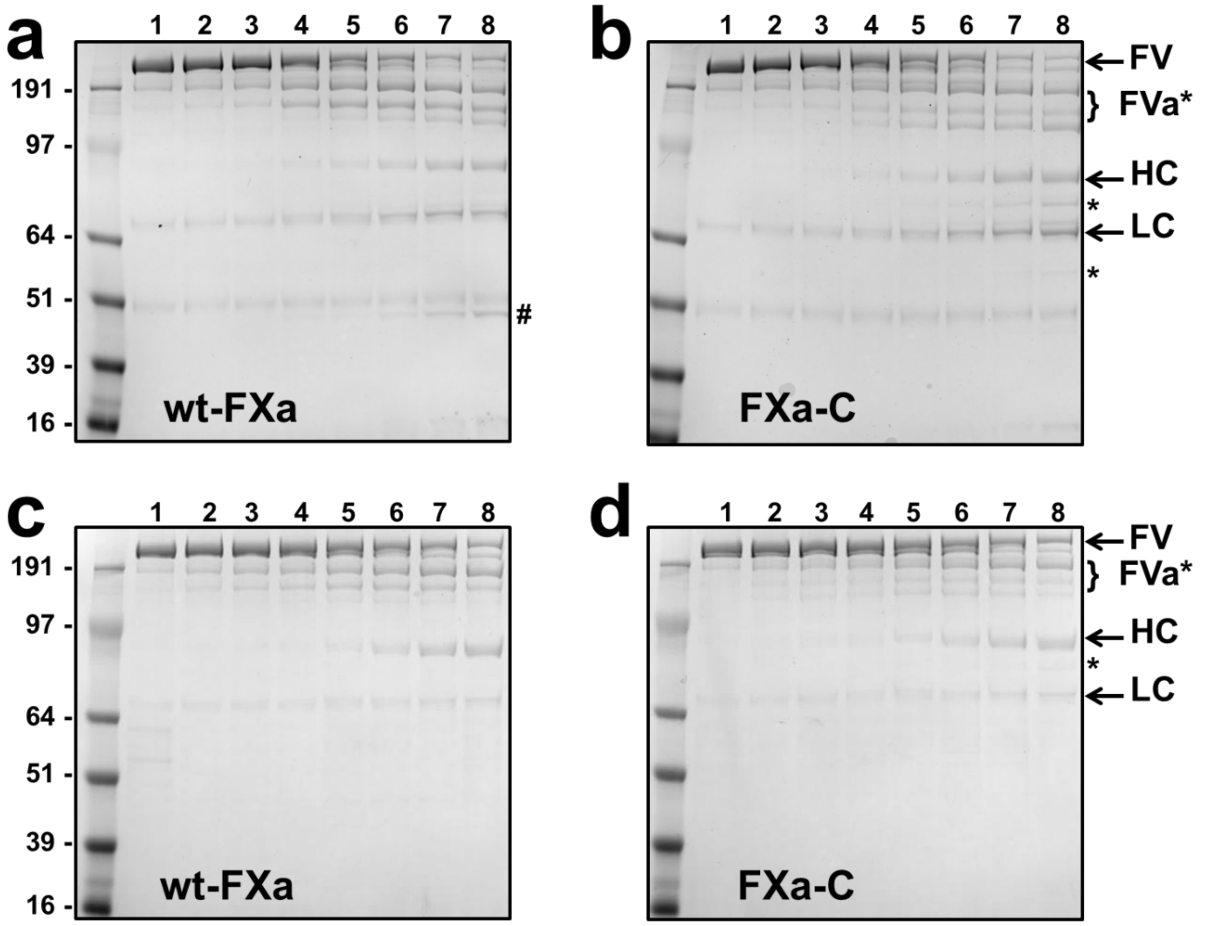

Supplementary Fig. 6. Activation of factor V by factor Xa variants. Plasma-derived (pd)-FV (400 nM) was incubated at 25°C either with increasing concentrations (0 – 100 nM) of wild-type FXa (wt-FXa; *a,c*) or FXa variant C (FXa-C; *b,d*) in the presence of 50 µM PCPS for 10 minutes (*a,b*) or with 5 nM wt-FXa or FXa-C in the presence of 25 µM PCPS for 1 – 60 minutes (*c,d*). Samples (3 µg per lane) were subjected to SDS-PAGE under reducing conditions using the MOPS buffer system and visualized by staining with Coomassie Brilliant Blue (CBB). (*a,b*) Lane 1: pd-FV, no FXa; lanes 2-8: pd-FV, wt-FXa or FXa-C at 0.5, 1, 5, 10, 20, 50, and 100 nM. (*c,d*) Lane 1: pd-FV, no FXa; lanes 2-8: pd-FV, incubated for 1, 2, 4, 10, 20, 40, and 60 minutes with wt-FXa or FXa-C. The protein bands indicative of single chain uncleaved pd-FV (FV), partially activated FV (FVa*), FVa heavy chain (HC),

FVa light chain (LC), FV activation fragment specific to wt-FXa (#), and FV activation fragments specific to FXa-C (*) are indicated. The apparent molecular weights of the standards (kDa) are indicated. The data are representative of two independent experiments.

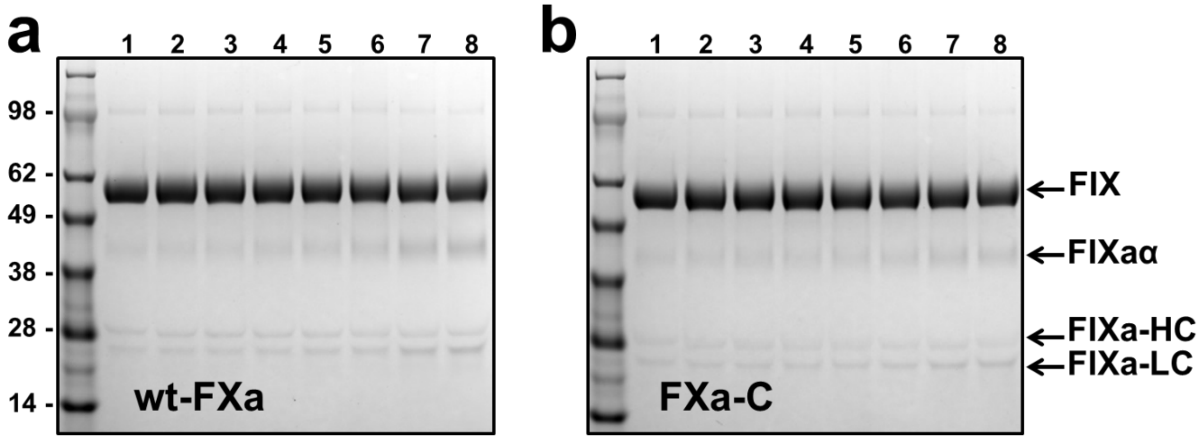

Supplementary Fig. 7. Cleavage of factor IX by factor Xa variants. Plasma-derived factor IX (2.7 µM; pd-FIX) was incubated for 1 – 60 minutes with 5 nM recombinant human wt-FXa (*a*) or FXa-C (*b*) in the presence of 50 µM PCPS at 25°C in assay buffer. Samples (3 µg per lane) were subjected to SDS-PAGE under reducing conditions using the MES buffer system and visualized by staining with CBB. Lane 1: pd-FIX, no FXa; lanes 2-8: pd-FIX, incubated for 1, 2, 4, 10, 20, 40, and 60 minutes with wt-FXa or FXa-C. The protein bands corresponding to full-length pd-FIX, partially activated FIX (FIXaα; 45 kDa), the heavy chain of fully activated pd-FIX (FIXa-HC; 28 kDa), or the light chain of activated pd-FIX (FXIa-LC; 17 kDa) are indicated. The apparent molecular weights of the standards (kDa) are indicated. The data suggest that both FXa variants are capable of partially activating FIX into FIXaα (≥40 minutes), and wt-FXa may be slightly more efficient in doing so. FIXaα is known to display catalytic activity toward synthetic substrates only and does not have clotting activity. The data represents one experiment.

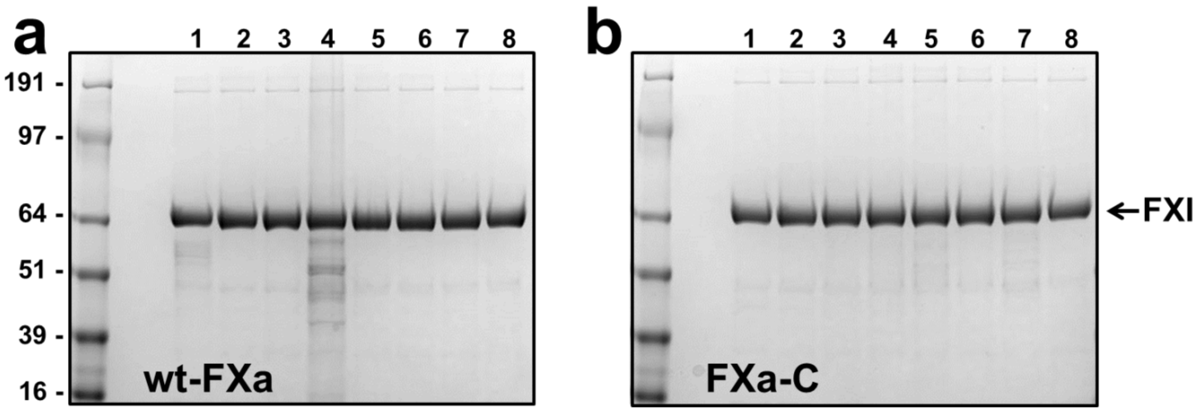

Supplementary Fig. 8. Cleavage of factor XI by factor Xa variants. Plasma-derived factor XI (0.9 µM; pd-FXI) was incubated for 1 – 60 minutes with 5 nM recombinant human wt-FXa (*a*) or FXa-C (*b*) in the presence of 50 µM PCPS at 25°C in assay buffer. Samples (3 µg per lane) were subjected to SDS-PAGE under reducing conditions using the MOPS buffer system and visualized by staining with CBB. Lane 1: pd-FXI, no FXa; lanes 2-8: pd-FXI, incubated for 1, 2, 4, 10, 20, 40, and 60 minutes with wt-FXa or FXa-C. The protein bands corresponding to full-length pd-FXI are indicated. The apparent molecular weights of the standards (kDa) are indicated. Conversion of the 80 kDa FXI zymogen subunit to the 50 and 30 kDa heavy and light chains of FXIa was not observed. The data represents one experiment.

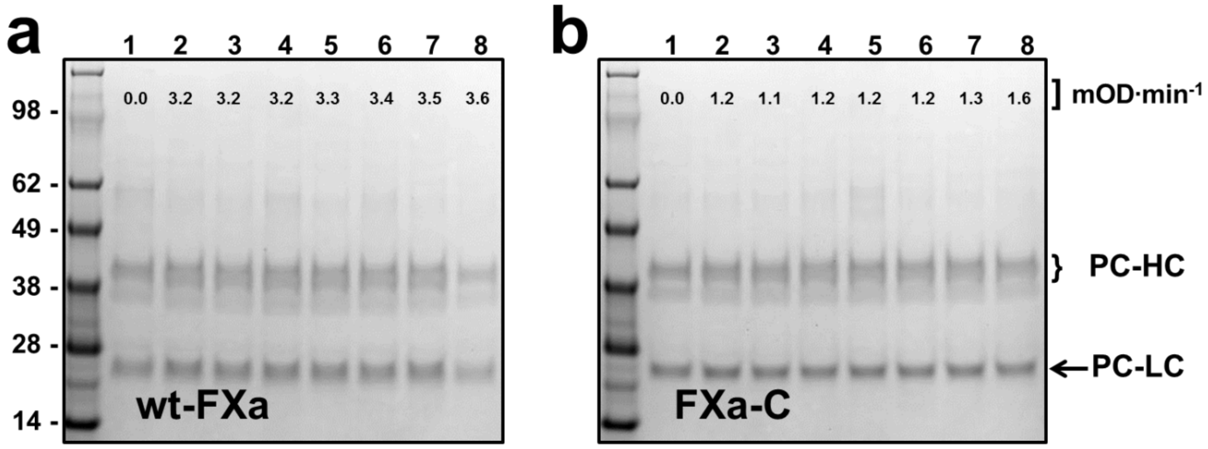

Supplementary Fig. 9. Cleavage of protein C by factor Xa variants. Plasma-derived protein C (2.4 µM; pd-PC) was incubated for 1 – 60 minutes with 5 nM recombinant human wt-FXa (*a*) or FXa-C (*b*) in the presence of 50 µM PCPS at 25°C in assay buffer. Samples (3 µg per lane) were subjected to SDS-PAGE under reducing conditions using the MES buffer system and visualized by staining with CBB. Lane 1: pd-PC, no FXa; lanes 2-8: pd-PC, incubated for 1, 2, 4, 10, 20, 40, and 60 minutes with wt-FXa or FXa-C. The protein bands corresponding to the heavy chain of pd-PC (PC-HC; 41 kDa) and the light chain of pd-PC (PD-LC; 21 kDa) are indicated. The apparent molecular weights of the standards (kDa) are indicated. Conversion of the 41 kDa pd-PC heavy chain to the 35 kDa light chain of activated protein C was not observed. These findings were corroborated by assessment of peptidyl substrate S2366 (250 µM final) conversion (indicated as mOD min^{-1}) by time samples of pd-PC (66 nM final). No significant activation of pd-PC was observed given that the chromogenic activity in samples 2 – 8 corresponded to the conversion of S2366 by 5 nM of either wt-FXa or FXa-C. The data represents one experiment.

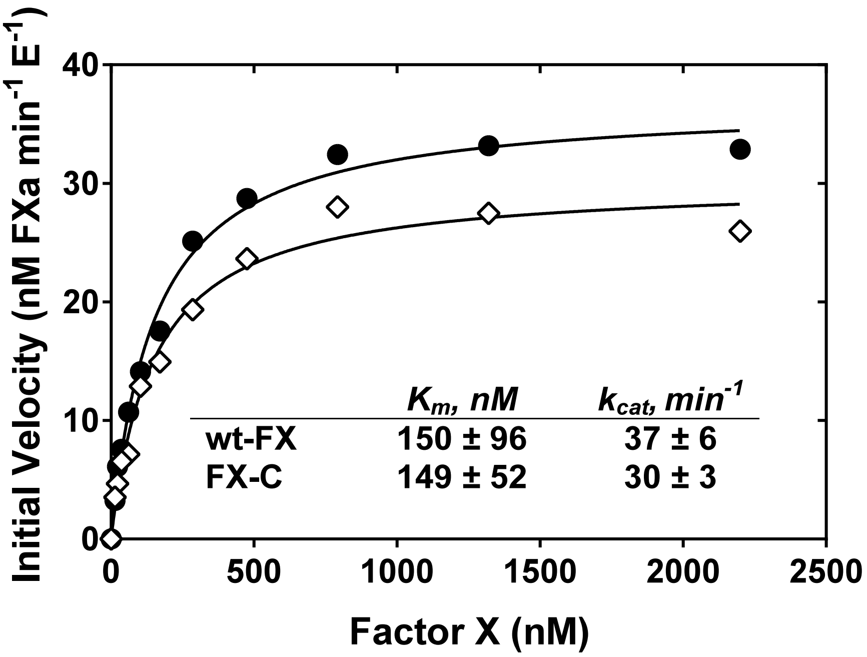

Supplementary Fig. 10. Activation of factor X variants via the intrinsic activation pathway. The rate of FX (13 – 2200 nM) activation for wild-type (wt-FX; closed circles) or variant C (FX-C; open diamonds) by the intrinsic FVIIIa/FIXa tenase complex (PCPS, 20 µM; FIXa, 0.5 nM; FVIIIa, 5 nM) was determined as described in 'Materials'. Individual data points represent the average of two independent experiments. The lines were drawn by fitting the data to the Michaelis-Menten equation using non-linear regression, and the obtained values for K_m and k_{cat} ± 1 standard deviation of the induced fit are shown in the inset.

Supplementary Table 1. Factor Xa-initiated thrombin generation in factor X-depleted plasma in the absence or presence of apixaban.

	pd-FXa	wt-FXa	FXa-A	FXa-B	FXa-C
No inhibitor					
**Lag time** *(min)*	0.14 ± 0.05	0.33 ± 0.06	2.28 ± 0.77	1.85 ± 0.44	0.42 ± 0.08
**Time to peak** *(min)*	0.74 ± 0.05	1.03 ± 0.10	3.75 ± 1.15	3.57 ± 0.99	1.30 ± 0.09
**Peak height** *(nM)*	938 ± 121	836 ± 11	625 ± 144	512 ± 170	827 ± 29
**Velocity index** *(nM* min^{-1}*)*	1565 ± 201	1203 ± 97	473 ± 228	323 ± 156	937 ± 25
**ETP** *(nM min)*	2573 ± 264	2667 ± 175	2313 ± 396	1979 ± 717	2792 ± 86
2 μM apixaban					
**Lag time** *(min)*	4.43 ± 1.71	5.22 ± 1.43	3.29 ± 2.08	2.02 ± 0.94	0.47 ± 0.12
**Time to peak** *(min)*	8.14 ± 2.63	8.82 ± 1.81	5.75 ± 3.41	4.07 ± 1.78	1.42 ± 0.20
**Peak height** *(nM)*	278 ± 128	272 ± 54	490 ± 214	435 ± 86	818 ± 40
**Velocity index** *(nM* min^{-1}*)*	87 ± 62	77 ± 22	289 ± 228	243 ± 113	868 ± 102
**ETP** *(nM min)*	1845 ± 296	2031 ± 187	2059 ± 375	1824 ± 442	2823 ± 95

Thrombin generation was measured for 60 minutes at 37°C in FX-depleted plasma supplemented with 20 μM PCPS. Thrombin generation was initiated by the addition of 5 nM plasma-derived human FXa (pd-FXa), recombinant wild-type human FXa (wt-FXa), or chimeric FXa-A, FXa-B, or FXa-C premixed with HBS (no inhibitor) or apixaban (2 μM) and supplemented with $CaCl_2$ and a thrombin fluorogenic substrate as detailed in 'Materials'. All values represent averages ± 1 standard deviation obtained from at least three independent experiments.

Supplementary Table 2. Tissue factor-initiated thrombin generation in factor X-depleted plasma spiked with factor X variants.

	no inhibitor	2.0 µM APX	6.0 µM APX	0.6 µM EDX	2.0 µM EDX
wt-FX					
**Lag time** *(min)*	2.23 ± 0.27	10.59 ± 1.51	17.77 ± 4.05	11.95 ± 1.27	22.86 ± 5.54
**Time to peak** *(min)*	5.12 ± 0.65	49.57 ± 1.81	51.82 ± 0.52	37.14 ± 8.60	45.70 ± 9.59
**Peak height** *(nM)*	86 ± 25	8 ± 2	4 ± 1	13 ± 3	7 ± 2
**Velocity index** *(nM min^{-1})*	31 ± 13	<1	<1	<1	<1
**ETP** *(nM min)*	646 ± 88	NA	NA	NA	NA
FX-C					
**Lag time** *(min)*	2.98 ± 0.30	3.00 ± 0.22	3.25 ± 0.22	3.43 ± 0.30	4.16 ± 0.54
**Time to peak** *(min)*	7.32 ± 0.73	7.67 ± 0.50	8.17 ± 0.50	8.53 ± 0.86	10.34 ± 1.25
**Peak height** *(nM)*	93 ± 18	83 ± 12	86 ± 14	85 ± 22	62 ± 18
**Velocity index** *(nM min^{-1})*	22 ± 6	18 ± 4	18 ± 4	17 ± 6	10 ± 4
**ETP** *(nM min)*	806 ± 99	776 ± 30	789 ± 43	846 ± 131	722 ± 70

Thrombin generation (TG) was measured for 60 minutes at 37°C in FX-depleted plasma supplemented with 2 pM tissue factor (TF), 20 µM PCPS, and either 1 U ml^{-1} of wt-FX (7 µg ml^{-1}) or FX-C (15 µg ml^{-1}). Thrombin generation was initiated with $CaCl_2$ and a thrombin fluorogenic substrate as detailed in 'Materials'. Experimental data was obtained in the absence or presence of either 2 µM or 6 µM apixaban (APX), or 0.6 µM or 2 µM edoxaban (EDX). All values represent averages ± 1 standard deviation obtained from at least three independent experiments. NA, not applicable: for these experiments thrombin generation was insufficient, precluding an accurate assessment of the ETP.

Supplementary Table 3. Tissue factor-initiated thrombin generation parameters in normal pooled plasma spiked with factor X-C and apixaban

	NPP	APX + 5 µg ml^{-1}	APX + 10 µg ml^{-1}	APX + 20 µg ml^{-1}	APX + 40 µg ml^{-1}	+ 40 µg ml^{-1}
2 pM TF						
**Lag time** ***(min)***	3.27 ± 0.16	4.45 ± 0.08	3.98 ± 0.06	3.69 ± 0.19	3.89 ± 0.20	3.25 ± 0.25
**Time to peak** ***(min)***	6.65 ± 0.28	12.88 ± 0.38	11.44 ± 0.31	10.35 ± 0.63	10.35 ± 0.51	8.58 ± 0.63
**Peak height** ***(nM)***	96 ± 22	50 ± 20	68 ± 17	77 ± 11	84 ± 11	87 ± 2
**Velocity index** ***(nM min^{-1})***	29 ± 6	6 ± 2	9 ± 2	12 ± 2	13 ± 3	16 ± 1
**ETP** ***(nM min)***	731 ± 127	719 ± 246	798 ± 191	740 ± 96	722 ± 45	696 ± 27
6 pM TF						
**Lag time** ***(min)***	1.94 ± 0.15	3.04 ± 0.06	2.56 ± 0.10	2.35 ± 0.12	2.27 ± 0.04	2.08 ± 0.14
**Time to peak** ***(min)***	4.10 ± 0.23	7.54 ± 0.56	6.10 ± 0.42	5.39 ± 0.30	5.35 ± 0.29	4.67 ± 0.14
**Peak height** ***(nM)***	171 ± 23	120 ± 36	143 ± 26	168 ± 22	176 ± 16	190 ± 12
**Velocity index** ***(nM min^{-1})***	79 ± 9	27 ± 8	40 ± 8	55 ± 6	57 ± 6	74 ± 5
**ETP** ***(nM min)***	792 ± 100	864 ± 268	839 ± 204	849 ± 118	883 ± 86	868 ± 25

Thrombin generation (TG) was measured for 60 minutes at 37°C in normal pooled plasma supplemented with 2 or 6 pM tissue factor (TF), 20 µM PCPS, and 5 – 40 µg ml^{-1} FX-C in the presence of 2 µM apixaban (APX). Thrombin generation was initiated with $CaCl_2$ and a thrombin fluorogenic substrate as detailed in 'Materials'. All values represent averages ± 1 standard deviation obtained from at least three independent experiments.

Supplementary References

1 Bos, M. H. & Camire, R. M. Procoagulant adaptation of a blood coagulation prothrombinase-like enzyme complex in australian elapid venom. *Toxins* **2**, 1554-1567, doi:10.3390/toxins2061554 (2010).
